# Serum Albumin as a Predictor of Pulmonary Embolism Severity: A Retrospective Study From Buraydah, Saudi Arabia

**DOI:** 10.7759/cureus.111821

**Published:** 2026-06-30

**Authors:** Mohammad H Alhassoon, Omar K Alsaawi, Nawaf H Alharbi, Khalid S Alrashdi, Rayan M Alharbi

**Affiliations:** 1 Pulmonology, King Fahad Specialist Hospital, Buraydah, SAU; 2 Internal Medicine, King Fahad Specialist Hospital, Buraydah, SAU

**Keywords:** hypoalbuminemia, massive pe, mortality, pesi score, prognostic biomarkers, pulmonary embolism, risk stratification, serum albumin

## Abstract

Background

Pulmonary embolism (PE) presents with a wide spectrum of severity, and the early identification of high-risk patients is essential for improving outcomes. Serum albumin has emerged as a potential prognostic biomarker for cardiovascular and thromboembolic diseases; however, its relationship with PE severity and clinical risk scores remains unclear. This study aimed to evaluate the association between serum albumin levels and PE severity, including massive PE, Pulmonary Embolism Severity Index (PESI) scores, and mortality in a tertiary hospital cohort.

Methodology

This retrospective study was conducted at King Fahad Specialist Hospital in Saudi Arabia between 2022 and 2025 and included 166 adult patients diagnosed with PE. Data on clinical characteristics, comorbidities, hemodynamic parameters, albumin values, troponin values, echocardiographic findings, CT pulmonary angiography, and outcomes were collected. The patients were categorized into five classes according to their PESI score. Furthermore, patients were categorized into massive and submassive/non-massive PE groups. Multivariate logistic regression was used to identify independent predictors of massive PE. Correlation analysis was used to assess the relationship between albumin and the PESI score, and receiver operating characteristic (ROC) analysis was used to evaluate the discriminatory performance of albumin for predicting massive PE.

Results

The mean age was 57.1 ± 19.4 years, and 93 (56.0%) patients were female. Massive PE was observed in 25 patients (15.1%). Hypoalbuminemia independently predicted massive PE (adjusted odds ratio (AOR) = 13.615, 95% confidence interval = 3.070-60.385; p = 0.001). Right ventricular (RV) strain (AOR = 9.781, p < 0.001) and prior stroke (AOR = 5.340, p = 0.009) were also significant predictors. There was a significant inverse correlation between albumin levels and PESI scores (r = -0.382, p < 0.001). The ROC analysis yielded an AUC of 0.628 (p = 0.005), with an optimal cutoff of 31.9 g/L (sensitivity = 84.8%; specificity = 58%). Mortality was significantly associated with albumin levels (p = 0.035).

Conclusions

The serum albumin level is a biomarker associated with PE severity, hemodynamic compromise, and adverse outcomes. Hypoalbuminemia independently predicted massive PE and showed a significant inverse correlation with PESI score, underscoring its value in risk stratification. The albumin level was also significantly associated with mortality, suggesting its broader prognostic relevance in acute PE. In models that assess multiple parameters, albumin demonstrated an independent association with PE severity alongside other clinical variables. These results need to be confirmed in prospective multicenter studies, and the mechanistic role of albumin in PE severity and mortality needs to be defined.

## Introduction

Pulmonary embolism (PE) is a life-threatening condition caused by blood clots that block arteries in the lungs. These clots often start as deep vein thrombosis in the legs. PE is the third leading cause of cardiovascular death worldwide after heart attack and stroke [1]. Symptoms can vary from being asymptomatic to having sudden cardiac arrest, depending on the clot size, the patient’s heart and lung health, and other medical issues. Fast and accurate diagnosis, along with proper risk assessment, are key to choosing appropriate treatment and improving outcomes. The Pulmonary Embolism Severity Index (PESI) and its simplified version (sPESI) are tools that help assess the severity of PE and the risk of death [2]. However, better markers are required to improve outcome prediction. New, affordable, and easy-to-use biomarkers can help provide personalized care. In this context, the primary objective of this retrospective study was to assess the association between serum albumin levels and PE severity. The secondary objectives were to evaluate the relationship between serum albumin levels, PESI scores, and mortality.

Serum albumin is produced in the liver. It helps maintain the fluid balance in blood vessels and regulates inflammation. Low albumin levels, known as hypoalbuminemia, are associated with worse outcomes in several illnesses, including venous thromboembolism (VTE) and heart failure [3,4]. In PE, low albumin levels may indicate inflammation, issues with the blood vessel lining, or poor nutrition. These problems can increase the severity of PE and the risk of death [5,6].

Several studies have shown that albumin is a simple and affordable marker for assessing risk in patients [7-9]. Folsom et al. (2010) found that low serum albumin levels were associated with a higher risk of VTE [10], and Chi et al. (2019) confirmed this finding in very sick hospital patients [11]. Gotsman et al. (2019) also found that low albumin levels predicted lower survival in patients with chronic heart failure [3].

Furthermore, Omar et al. reported that patients with massive PE had lower average albumin levels than those with less severe PE. Albumin level was also a strong predictor of PE severity (area under the curve (AUC) = 0.750) [4]. Hoskin et al. (2020) reported that low albumin levels were associated with more complications and higher hospital death rates after acute PE [5]. Tanık et al. (2020) reported that low albumin levels were associated with worse long-term outcomes in patients with PE [6]. Liu et al. (2020) found that low preoperative albumin levels predicted a higher risk of death within 30 days of surgery in patients with PE [7]. Zamlout et al. conducted a longitudinal study following patients over time and found that low albumin levels were associated with higher sPESI scores, lower oxygen levels, and more deaths [8]. Huang et al. (2023) used both observational data and genetic analyses to show that low serum albumin levels can directly increase the risk of PE [9].

Researchers are also studying combined markers, and not just albumin levels alone. Özcan et al. (2022) found that the ratio of C-reactive protein to albumin (CRP/Alb) was strongly associated with PE severity and the risk of death [12]. Chang et al. (2025) introduced the platelet-to-albumin ratio to predict 28-day death rates in critically ill patients with PE using machine learning to improve accuracy [13].

Eraslan et al. and Ding et al. proposed red cell distribution width-to-albumin ratio (RDW/Alb) as a new prognostic marker. Both studies showed that a higher RDW/Alb was independently associated with increased mortality in acute PE [14,15]. Başyiğit et al. (2025) refined this idea by introducing the RDW-standard deviation-to-albumin ratio, which showed strong predictive value for six-month mortality [16].

Although there is growing global evidence that serum albumin is a useful prognostic marker for PE, there are few data on the Middle Eastern population. Hence, this study aims to contribute to the management and prevention of this life-threatening condition. By analyzing the clinical, laboratory, and radiological data, we can address whether albumin is a practical and accessible biomarker for improving PE outcome prediction in this region.

## Materials and methods

This study was designed as a single-center, retrospective observational analysis of conveniently admitted patients diagnosed with PE at King Fahad Specialist Hospital in Buraydah, Qassim, Saudi Arabia, between 2022 and 2025. Inclusion criteria comprised patients with a confirmed diagnosis of PE based on computed tomography pulmonary angiography (CTPA). Patients were excluded if they had conditions known to significantly affect serum albumin levels, including sepsis, active infections, or chronic inflammatory diseases, or if their medical records were incomplete. A total of 166 patients with PE were included in the study after application of the inclusion and exclusion criteria. Six patients were excluded, including two with no serum albumin level measured at admission, two with pneumonia, and two with urinary tract infection.

After obtaining approval from the hospital administration and institutional ethics committee, data were collected from patients’ medical records and organized in an Excel spreadsheet for analysis. The extracted data included demographic variables (age and sex) and comorbid conditions, such as diabetes mellitus, hypertension, ischemic heart disease, heart failure, chronic lung disease, previous stroke, atrial fibrillation, antiphospholipid syndrome, and other inherited or acquired thrombophilias, including antithrombin III deficiency, protein C deficiency, and protein S deficiency. In addition, information on prior malignancies and smoking status was recorded.

Clinical presentations at admission, including cough, dyspnea, hemoptysis, chest pain, and altered mental status, were also documented. The vital signs and oxygen saturation levels were recorded upon admission. Laboratory data, including serum albumin and troponin levels, were obtained, along with imaging findings from echocardiography and CTPA at the time of admission in the emergency department.

The PESI was calculated for all patients using the original validated scoring system, which incorporates age, sex, comorbidities (malignancy, heart failure, and chronic lung disease), and clinical parameters, including heart rate, systolic blood pressure, respiratory rate, temperature, oxygen saturation, and mental status. Based on the total score, patients were stratified into the following five PESI classes: Class I (≤65 points), Class II (66-85 points), Class III (86-105 points), Class IV (106-125 points), and Class V (≥125 points), corresponding to increasing severity and mortality risk [2]. Additionally, patients were categorized according to PE severity and were further categorized as having massive or submassive/non-massive PE. Massive PE was defined as sustained systolic blood pressure <90 mmHg for at least 15 minutes, requirement for vasopressor support, cardiac arrest, or persistent profound bradycardia associated with signs of shock. Submassive PE was defined as normotensive PE with evidence of right ventricular dysfunction on echocardiography or CTPA and/or elevated levels of cardiac biomarkers such as troponin. Non-massive PE was defined as PE without hemodynamic instability, right ventricular dysfunction, or elevated cardiac biomarker levels. Serum albumin levels were categorized as hypoalbuminemia and normal albumin levels, with hypoalbuminemia defined as a serum albumin concentration <35 g/L.

Statistical analysis

Data were analyzed using SPSS version 26 (IBM Corp., Armonk, NY, USA). Categorical variables are presented as numbers and percentages. For continuous variables, we reported the mean and standard deviation, as well as the median and range. We compared PE severity with the patients’ demographic and clinical characteristics using Fisher’s exact test, independent-sample t-test, and Kruskal-Wallis test. Multivariate regression analysis was used to identify significant predictors of massive PE. The multivariate logistic regression model was adjusted for age, sex, diabetes mellitus, and hypertension. The association between mortality and albumin levels was assessed using an independent-sample t-test. Furthermore, Pearson’s correlation coefficient was calculated to determine the correlation between albumin levels and PESI scores in patients with massive PE. For albumin, we performed receiver operating characteristic (ROC) analysis to identify the cut-off points for detecting significant results in patients with massive acute PE. Statistical significance was set at a p-value <0.05.

## Results

**Figure 1:**
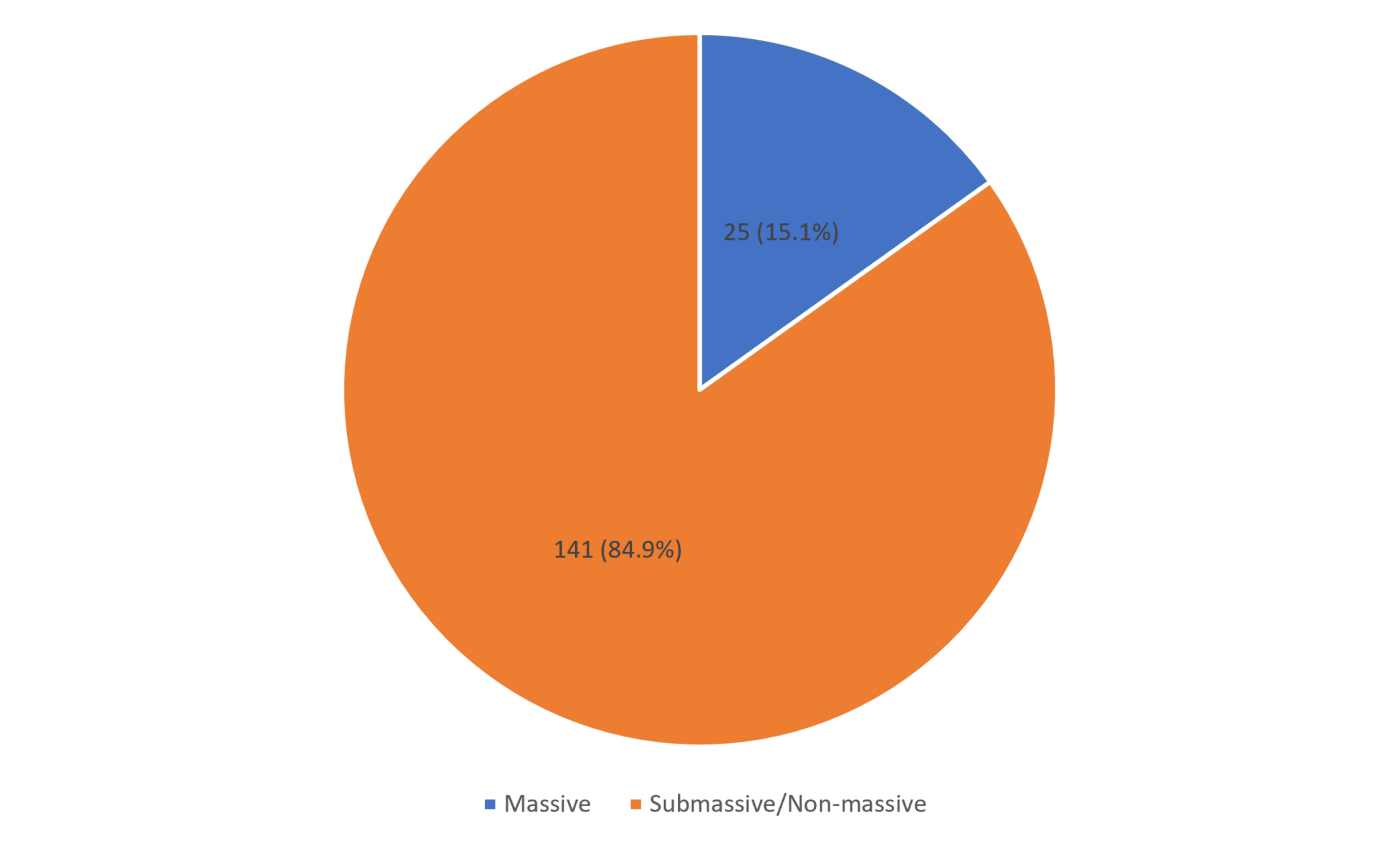
Frequency of massive pulmonary embolism.

This study included 166 patients with a mean age of 57.1 years (±19.4), of whom 93 (56.0%) were female (Table 1). The most common comorbidities were hypertension in 73 (44.0%) patients, diabetes in 61 (36.7%) patients, and previous stroke in 24 (14.5%) patients. The most common presenting symptom among the patients was dyspnea in 155 (93.4%) patients, followed by chest pain in 91 (54.8%) patients, and cough in 39 (23.5%) patients. Hemodynamic instability occurred in 25 (15.1%) patients. Right ventricular strain was observed in 84 (50.6%) patients. The mortality rate was 16.3% (N = 27). Overall, 80 (48.2%) patients had low albumin levels. The frequency of massive PE in this cohort was 15.1% (N = 25), whereas the remaining 84.9% (N = 141) had submassive or non-massive PE (Figure 1).

**Table 1:** Demographic and clinical characteristics of the patients in relation to PESI severity classes. Results are presented as numbers and percentages, N (%), as well as mean ± standard deviation. §: P-value was calculated using Fisher’s exact test; ^‡^: P-value was calculated using the chi-square test; ^†^: P-value was calculated using the one-way analysis of variance test; *: Significant at p-values <0.05; **: Highly significant at p-values <0.001. AMS = altered mental status; APS = antiphospholipid syndrome; LVEF = left ventricular ejection fraction; PE = pulmonary embolism; PESI = Pulmonary Embolism Severity Index; RV = right ventricular

Study variable	Overall, N (%) (n = 166)	PE severity class					Test statistics	P-value
		Class I, N (%) (n = 52)	Class II, N (%) (n = 38)	Class III, N (%) (n = 32)	Class IV, N (%) (n = 13)	Class V, N (%) (n = 31)		
Age in years (mean ± SD)^†^	57.1 ± 19.4	38.9 ± 10.6	58.3 ± 17.1	69.8 ± 15.1	62.8 ± 14.1	70.6 ± 15.9	F = 34.127	<0.001**
Gender^§^								
Male	73 (44.0%)	24 (46.2%)	13 (34.2%)	17 (53.1%)	6 (46.2%)	13 (41.9%)	Fisher’s exact test	0.603
Female	93 (56.0%)	28 (53.8%)	25 (65.8%)	15 (46.9%)	7 (53.8%)	18 (58.1%)		
Diabetes^‡^								
No	105 (63.3%)	44 (84.6%)	20 (52.6%)	17 (53.1%)	6 (46.2%)	18 (58.1%)	χ² = 15.460	0.002*
Yes	61 (36.7%)	8 (15.4%)	18 (47.4%)	15 (46.9%)	7 (53.8%)	13 (41.9%)		
Hypertension^‡^								
No	93 (56.0%)	44 (84.6%)	19 (50.0%)	9 (28.1%)	6 (46.2%)	15 (48.4%)	χ² = 29.171	<0.001**
Yes	73 (44.0%)	8 (15.4%)	19 (50.0%)	23 (71.9%)	7 (53.8%)	16 (51.6%)		
Ischemic heart disease^§^								
No	150 (90.4%)	51 (98.1%)	37 (84.4%)	27 (84.4%)	11 (84.6%)	24 (77.4%)	Fisher’s exact test	0.004*
Yes	16 (9.6%)	1 (1.9%)	1 (2.6%)	5 (15.6%)	2 (15.4%)	7 (22.6%)		
Heart failure^§^								
No	145 (87.3%)	52 (100%)	34 (89.5%)	24 (75.0%)	11 (84.6%)	24 (77.4%)	Fisher’s exact test	0.001*
Yes	21 (12.7%)	0	4 (10.5%)	8 (25.0%)	2 (15.4%)	7 (22.6%)		
Chronic kidney disease^§^								
No	149 (89.8%)	47 (90.4%)	34 (89.5%)	29 (90.6%)	12 (92.3%)	27 (87.1%)	Fisher’s exact test	0.987
Yes	17 (10.2%)	5 (9.6%)	4 (10.5%)	3 (9.4%)	1 (7.7%)	4 (12.9%)		
Old stroke^§^								
No	142 (85.5%)	51 (98.1%)	33 (86.8%)	28 (87.5%)	12 (92.3%)	18 (58.1%)	Fisher’s exact test	<0.001**
Yes	24 (14.5%)	1 (1.9%)	5 (13.2%)	4 (12.5%)	1 (7.7%)	13 (41.9%)		
Atrial fibrillation^§^								
No	153 (92.2%)	52 (100%)	38 (100%)	29 (90.6%)	11 (84.6%)	23 (74.2%)	Fisher’s exact test	<0.001**
Yes	13 (7.8%)	0	0	3 (9.4%)	2 (15.4%)	8 (25.8%)		
APS^§^								
No	163 (98.2%)	49 (94.2%)	38 (100%)	32 (100%)	13 (100%)	31 (100%)	Fisher’s exact test	0.356
Yes	3 (1.8%)	3 (5.8%)	0	0	0	0		
Other thrombophilia^§^								
No	165 (99.4%)	51 (98.1%)	38 (100%)	32 (100%)	13 (100%)	31 (100%)	Fisher’s exact test	1.000
Yes	1 (0.6%)	1 (1.9%)	0	0	0	0		
Chronic lung disease^§^								
No	148 (89.2%)	49 (94.2%)	34 (89.5%)	29 (90.6%)	10 (76.9%)	26 (83.9%)	Fisher’s exact test	0.313
Yes	18 (10.8%)	3 (5.8%)	4 (10.5%)	3 (9.4%)	3 (23.1%)	5 (16.1%)		
History of cancer^§^								
No	151 (91.0%)	52 (100%)	33 (86.8%)	30 (93.8%)	10 (76.9%)	26 (83.9%)	Fisher’s exact test	0.035*
Yes	15 (9.0%)	0	5 (13.2%)	2 (6.3%)	3 (23.1%)	5 (16.1%)		
Dyspnea^§^								
No	11 (6.6%)	4 (7.7%)	3 (7.9%)	1 (3.1%)	0	3 (9.7%)	Fisher’s exact test	0.798
Yes	155 (93.4%)	48 (92.3%)	35 (92.1%)	31 (96.9%)	13 (100%)	28 (90.3%)		
Cough^§^								
No	127 (76.5%)	39 (75.0%)	30 (78.9%)	25 (78.1%)	9 (69.2%)	24 (77.4%)	Fisher’s exact test	0.953
Yes	39 (23.5%)	13 (25.0%)	8 (21.1%)	7 (21.9%)	4 (30.8%)	7 (22.6%)		
Hemoptysis^§^								
No	148 (89.2%)	41 (78.8%)	35 (92.1%)	30 (93.8%)	12 (92.3%)	30 (96.8%)	Fisher’s exact test	0.100
Yes	18 (10.8%)	11 (21.2%)	3 (7.9%)	2 (6.3%)	1 (7.7%)	1 (3.2%)		
Chest pain^‡^								
No	75 (45.2%)	20 (38.5%)	12 (31.6%)	18 (56.3%)	6 (46.2%)	19 (61.3%)	χ² = 8.623	0.071
Yes	91 (54.8%)	32 (61.5%)	26 (68.4%)	14 (43.7%)	7 (53.8%)	12 (38.7%)		
AMS^§^								
Altered	27 (16.3%)	0	0	0	2 (15.4%)	25 (80.6%)	Fisher’s exact test	<0.001**
Normal	139 (83.7%)	52 (100%)	38 (100%)	32 (100%)	11 (84.6%)	6 (19.4%)		
Bradycardia^§^								
No	160 (96.4%)	51 (98.1%)	36 (94.7%)	32 (100%)	13 (100%)	28 (90.2%)	Fisher’s exact test	0.291
Yes	6 (3.6%)	1 (1.9%)	2 (5.3%)	0	0	3 (9.7%)		
Hemodynamic instability^§^								
No	141 (84.9%)	50 (96.2%)	34 (89.5%)	30 (93.8%)	10 (76.9%)	17 (54.8%)	Fisher’s exact test	<0.001**
Yes	25 (15.1%)	2 (3.8%)	4 (10.5%)	2 (6.3%)	3 (23.1%)	14 (45.2%)		
LVEF^§^								
Not done	12 (7.2%)	4 (7.7%)	2 (5.3%)	1 (3.1%)	0	5 (16.1%)	Fisher’s exact test	0.001*
Normal	118 (71.1%)	46 (88.5%)	30 (78.9%)	20 (62.5%)	9 (69.2%)	13 (41.9%)		
<40%	9 (5.4%)	2 (3.8%)	1 (2.6%)	1 (3.1%)	1 (7.7%)	4 (12.9%)		
40–49%	27 (16.3%)	0	5 (13.2%)	10 (31.3%)	3 (23.1%)	9 (29.0%)		
RV strain^§^								
No	82 (49.4%)	33 (63.5%)	15 (39.5%)	18 (56.3%)	3 (23.1%)	13 (41.9%)	Fisher’s exact test	0.033*
Yes	84 (50.6%)	19 (36.5%)	23 (60.5%)	14 (43.7%)	10 (76.9%)	18 (58.1%)		
Mortality^§^								
No	139 (83.7%)	50 (96.2%)	33 (86.8%)	22 (68.8%)	12 (92.3%)	22 (71.0%)	Fisher’s exact test	0.002*
Yes	27 (16.3%)	2 (3.8%)	5 (13.2%)	10 (31.3%)	1 (7.7%)	9 (29.0%)		
Low albumin level^‡^								
No	86 (51.8%)	23 (44.2%)	20 (52.6%)	20 (62.5%)	8 (61.5%)	9 (29.0%)	χ² = 8.736	0.067
Yes	80 (48.2%)	29 (55.8%)	18 (47.4%)	12 (37.5%)	5 (38.5%)	22 (71.0%)		
PE level^§^								
Massive	25 (15.1%)	2 (3.8%)	4 (10.5%)	2 (6.3%)	3 (23.1%)	14 (45.2%)	Fisher’s exact	<0.001**
Submassive/Non-massive	141 (84.9%)	50 (96.2%)	34 (89.5%)	30 (93.8%)	10 (76.9%)	17 (54.8%)		

There were significant differences in several demographic and clinical features between PESI severity classes. Age increased progressively with each higher PESI class (p < 0.001), and patients in Class I were significantly younger than those in Classes III-V. Diabetes was significantly more common with increasing severity (p = 0.002), and patients with diabetes were more often in Classes II-V than in Class I. Hypertension was also significantly associated with higher PESI classes (p < 0.001) and was more prevalent in Classes III-V. Similarly, ischemic heart disease (p = 0.004) and heart failure (p = 0.001) were over-represented in the higher-risk classes, particularly in Classes IV and V. There was a significant gradient (p < 0.001) in the history of stroke, with the highest proportion of patients with prior cerebrovascular events in Class V. Atrial fibrillation was significantly more frequent in severe classes (p < 0.001), particularly in Class V, affecting one-quarter of the patients.

In addition, there was a significant association with cancer history (p = 0.035) and was more frequent in Classes IV and V. Altered mental status had one of the steepest gradients (p < 0.001), absent in Classes I-III but present in 80.6% of patients in Class V. Hemodynamic instability increased significantly with severity (p < 0.001), with almost half of the Class V patients being affected compared to only 1.9% in Class I. There was also a significant difference in the echocardiographic findings (p = 0.001) with reduced ejection fraction, and abnormal findings were more common in the higher classes of PESI. Right ventricular strain was significantly associated with increased severity (p = 0.033), particularly in Classes IV and V. The highest PESI class had a significantly higher mortality rate (p = 0.002). Finally, massive PE was highly associated with severe PESI classes (p < 0.001), with almost half of Class V patients classified as having massive PE.

As shown in Table 2, the mean systolic blood pressure was 121.7 mmHg, and the mean diastolic blood pressure was 74.2 mmHg. The mean heart rate and respiratory rate were 98.5 beats/minute and 20.6 breaths/minute, respectively. The mean SpO₂ was 89.7%. The mean troponin and albumin levels were 0.21 ng/mL and 34.3 g/L, respectively. Across the PE severity classes, we found significant differences in clinical and laboratory parameters. Systolic blood pressure decreased progressively with increasing severity (p < 0.001), with the lowest values observed in Class V, indicating worsening hemodynamic compromise. Diastolic blood pressure showed a similar pattern, declining significantly in the high-risk classes (p = 0.001). Oxygen saturation demonstrated a strong inverse gradient (p < 0.001), falling steadily from Class I to Class V, which was consistent with increased hypoxemia in severe PE. Albumin levels also declined significantly across the PESI classes (p < 0.001), with the lowest concentrations observed in Class V. The heart rate, respiratory rate, temperature, and troponin levels did not differ significantly across the PESI classes (p > 0.05).

**Table 2:** Association between metabolic characteristics and PESI severity classes using the one-way analysis of variance. Results are presented as means ± standard deviations. §: P-value has been calculated using the one-way analysis of variance test; *: Significant at p-values <0.05; **: Highly statistically significant at p-values <0.001. DBP = diastolic blood pressure; HR = heart rate; PESI = Pulmonary Embolism Severity Index; RR = respiratory rate; SBP = systolic blood pressure; SpO₂ = peripheral oxygen saturation

Variable	Overall, mean ± SD (n = 166)	PESI severity score					F-test	P-value^§^
		Class I, mean ± SD (n = 52)	Class II, mean ± SD (n = 38	Class III, mean ± SD (n = 32)	Class IV, mean ± SD (n = 13)	Class V, mean ± SD (n = 31)		
SBP (mmHg)	121.7 ± 22.8	123.3 ± 13.9	129.1 ± 21	129.9 ± 20.7	119.4 ± 22.6	103 ± 28.3	8.713	<0.001**
DBP (mmHg)	74.2 ± 13.4	75.1 ± 9.17	76.2 ± 13.9	78.4 ± 10.9	76.0 ± 9.51	65.3 ± 18.0	5.006	0.001*
HR (beats/minute)	98.5 ± 20.9	93.1 ± 13.7	97.0 ± 22.2	101.3 ± 19.3	106.4 ± 18.6	102.6 ± 28.9	1.821	0.127
RR (breaths/minute)	20.6 ± 6.08	19.2 ± 4.17	19.9 ± 7.28	20.7 ± 4.90	22.0 ± 4.65	23.4 ± 8.42	1.976	0.102
Temperature (°C)	36.9 ± 0.21	36.9 ± 0.27	36.9 ± 0.19	36.9 ± 0.16	37.0 ± 0.17	36.9 ± 0.21	0.471	0.757
SpO₂	89.7 ± 9.14	95.1 ± 2.64	91.5 ± 6.89	88.3 ± 9.59	84.3 ± 3.99	77.4 ± 14.7	8.681	<0.001**
Troponin (ng/mL)	0.21 ± 1.12	0.003 ± 0.02	0.19 ± 0.56	0.08 ± 0.26	0.13 ± 0.24	0.77 ± 2.53	1.917	0.112
Albumin (g/L)	34.3 ± 5.42	37.3 ± 4.40	34.4 ± 5.21	32.9 ± 5.76	33.1 ± 4.40	31.2 ± 4.99	8.510	<0.001**

Several clinical factors independently predicted massive PE after adjusting for age, sex, diabetes, and hypertension (Table 3). A history of stroke increased the likelihood of massive PE by more than five times (adjusted odds ratio (AOR) = 5.340, 95% confidence interval (CI) = 1.521-18.748, p = 0.009). Right ventricular strain also increased the odds by nearly 10-fold (AOR = 9.781, 95% CI = 2.748-34.881, p < 0.001). Low albumin levels were also associated with a 13-fold increase in risk (AOR = 13.615, 95% CI = 3.070-60.385, p = 0.001). In-hospital mortality was also significantly associated with massive PE (AOR = 2.733, 95% CI = 1.003-7.448, p = 0.049). PESI classification further stratified risk: compared with Class I, patients in Class II had dramatically higher odds of massive PE (AOR = 68.60, 95% CI = 8.949-525.9, p < 0.001), followed by Class III (AOR = 13.20, 95% CI = 2.971-58.67, p = 0.001) and Class IV (AOR = 12.39, 95% CI = 2.340-65.69, p = 0.003). PESI Class V showed an elevated but statistically non-significant association (AOR = 3.734, 95% CI = 0.759-18.369, p = 0.105).

**Table 3:** Multivariate logistic regression analysis to determine the independent significant predictors of massive PE (n = 166). Adjusted for age, gender, diabetes, and hypertension. *: Significant at p-values <0.05; **: Highly statistically significant at p-values <0.001. AOR = adjusted odds ratio; CI = confidence interval; RV = right ventricular; PE = pulmonary embolism; PESI = Pulmonary Embolism Severity Index

Factor	AOR	95% CI	P-value
Old stroke			
No	Ref		
Yes	5.340	1.521–18.748	0.009*
PESI category			
Class I: Very low risk	Ref		
Class II: Low risk	68.60	8.949–525.9	<0.001**
Class III: Intermediate risk	13.20	2.971–58.67	0.001*
Class IV: High risk	12.39	2.340–65.69	0.003*
Class V: Very high risk	3.734	0.759–18.369	0.105
Mortality			
No	Ref		
Yes	2.733	1.003–7.448	0.049*
RV strain			
No	Ref		
Yes	9.781	2.748–34.881	<0.001**
Low albumin			
No	Ref		
Yes	13.615	3.070–60.385	0.001*

As shown in Table 4, serum albumin level demonstrated a statistically significant association with mortality among the 166 patients (χ² = 4.450, p = 0.035). Among the 139 survivors, 67 (48.2%) had low albumin levels (<35 g/L) and 72 (51.8%) had normal albumin levels. In contrast, among the 27 patients who died, low albumin levels were present in 19 (70.4%) patients, representing a substantially higher proportion compared with 8 (29.6%) patients who had normal albumin levels. This pattern indicates that mortality was more common among patients with hypoalbuminemia, supporting the role of low albumin levels as a potential marker of physiological compromise and increased vulnerability.

**Table 4:** Association between mortality and albumin level (n = 166). Results are presented as numbers and percentages, N (%). ^§^: P-value was calculated using the chi-square test; *: Significant at p-values <0.05.

Factor	Mortality		Chi-square	P-value^§^
	No, N (%) (n = 139)	Yes, N (%) (n = 27)		
Low albumin				
Low (<35 g/L)	67 (48.2%)	19 (70.4%)	4.450	0.035*
Normal (≥35 g/L)	72 (51.8%)	8 (29.6%)		

**Figure 2:**
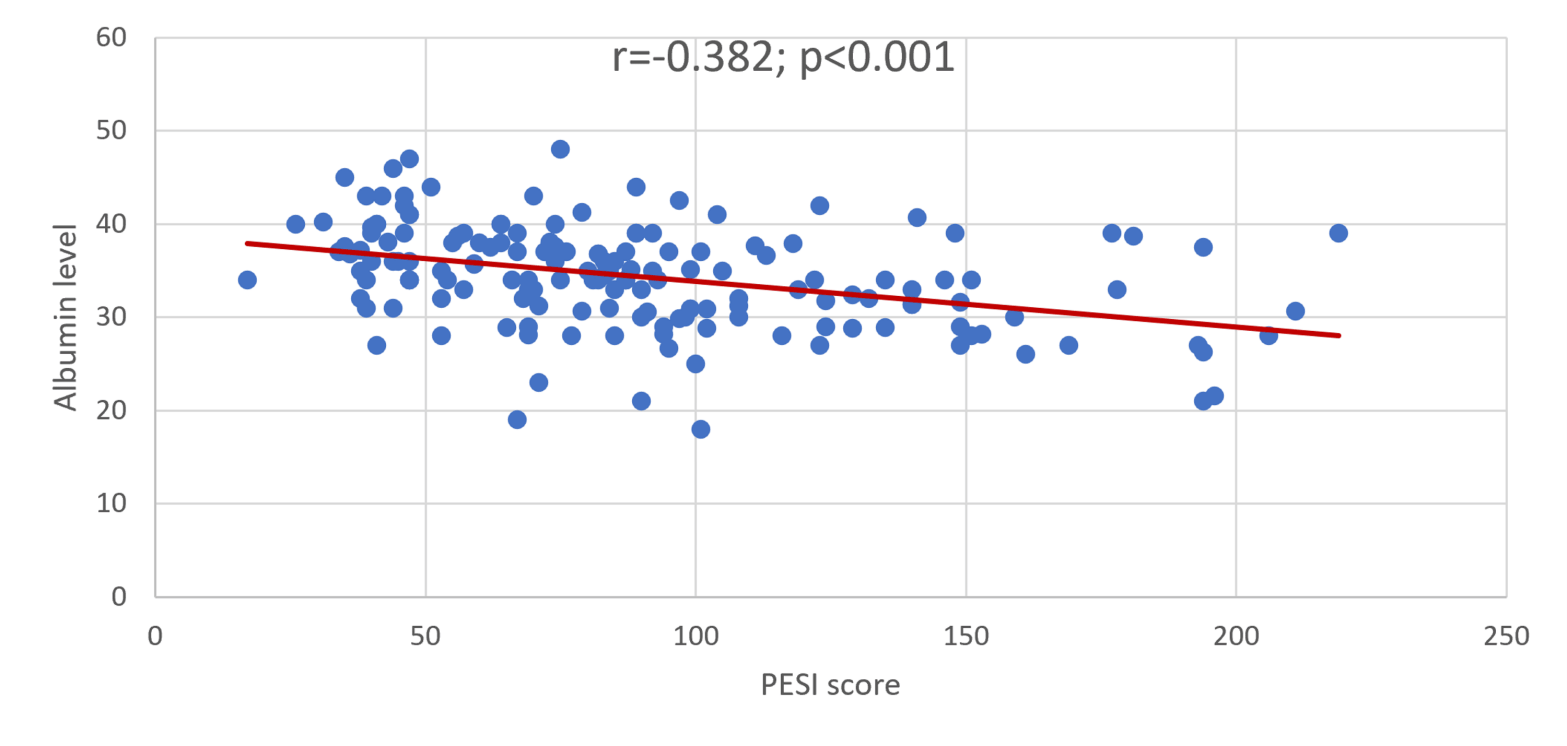
Pearson correlation coefficient between the Pulmonary Embolism Severity Index (PESI) score and albumin level.

Figure 2 demonstrates a moderate, statistically significant inverse correlation between PESI score and serum albumin level (r = -0.382, p < 0.001). The downward-sloping regression line indicates that higher PESI scores, which reflect a greater predicted mortality risk, are associated with lower albumin concentrations.

**Figure 3:**
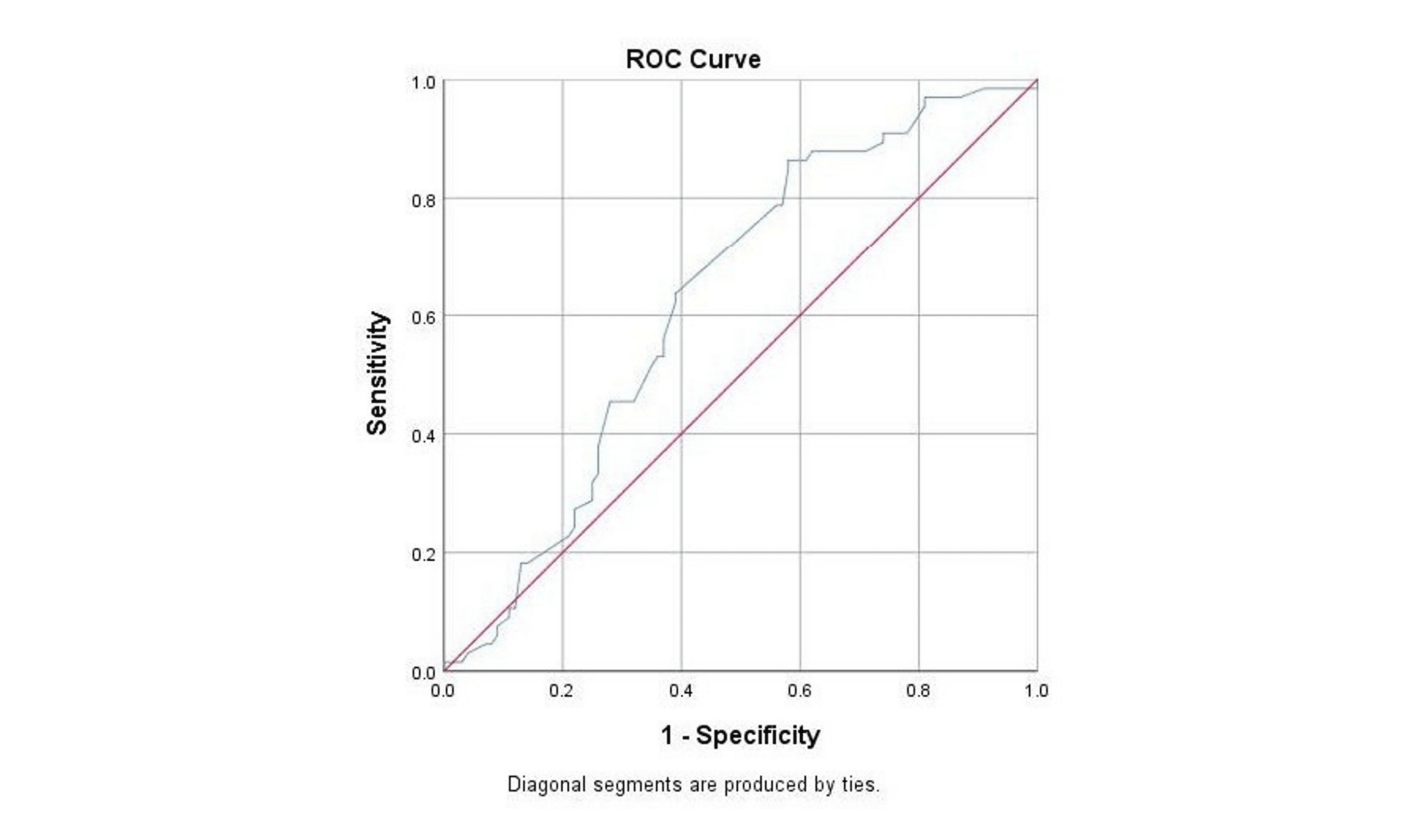
Receiver operating characteristics (ROC) analysis determining the optimal cutoff point of massive pulmonary embolism in relation to albumin level.

ROC analysis for albumin (Figure 3) showed an AUC of 0.628 (p = 0.005). A cutoff of 31.9 g/L gave a sensitivity of 84.8% and a specificity of 58%.

## Discussion

Among this cohort of 166 patients with PE, we found distinct demographic, clinical, and hemodynamic gradients across PESI severity classes, in line with the progressive physiological compromise reported in the seminal literature on PE. With each higher PESI class, there was a marked increase in age, which is consistent with the central role of advanced age in the original PESI derivation model of Aujesky et al. [2]. Comorbidities such as diabetes, hypertension, ischemic heart disease, heart failure, and prior stroke were overrepresented in Classes III-V, which is consistent with the known evidence that cardiometabolic disease and a history of cerebrovascular disease increase the morbidity and mortality of PE [1,2]. The high prevalence of atrial fibrillation and history of cancer in the higher classes further suggests that thromboembolic risk factors tend to cluster in patients with advanced systemic diseases.

Clinical instability markers also showed strong severity-dependent patterns. Altered mental status was absent in Classes I-III, but occurred in >80% of patients in Class V and reflects the profound hypoperfusion and decreased cerebral oxygenation described in the pathophysiology of massive PE [1]. PESI class was associated with a large increase in hemodynamic instability, affecting nearly 50% of Class V patients, which is consistent with the transition from compensated right ventricular strain to overt right ventricular failure in high-risk PE. Echocardiographic abnormalities such as reduced ejection fraction and right ventricular strain were significantly more prevalent in classes IV and V, supporting the prognostic significance of right ventricular dysfunction, as underlined in previous studies [4-6]. The mortality rate was significantly higher in the highest PESI class, confirming the discriminative performance of PESI in identifying patients with an immediate risk of clinical deterioration.

This study demonstrated a strong association between serum albumin levels and PE severity. Hypoalbuminemia was common, observed in 80 (48.2%) patients, and was an independent predictor of massive PE with a 13-fold increased risk. Albumin levels decreased progressively across PESI classes. Mortality was significantly higher in patients with low albumin. These findings support the concept that albumin reflects the chronic physiological reserve and acute systemic stress. This interpretation is consistent with the cardiovascular evidence that low albumin levels are associated with inflammation, endothelial dysfunction, and adverse outcomes of chronic heart failure [3]. Our findings are consistent with those of Omar et al. and Hoskin et al., who reported that hypoalbuminemia is associated with more severe PE and worse outcomes [4,5], and Tanık et al., who demonstrated that albumin levels <35 g/L predict long-term mortality [6]. Liu et al. showed that albumin combined with inflammatory markers predicted the 30-day mortality after PE surgery [7].

However, not all studies have reported consistent associations. Zamlout et al. found no significant correlation between albumin and PE severity and hypothesized that hypoalbuminemia may reflect chronic illness rather than acute PE severity [8]. Differences in the comorbidity burden, timing of albumin measurement, and definition of severity are likely to explain these discrepancies. In our cohort, chronic diseases, such as diabetes and hypertension, were common in higher PESI classes and may have contributed to baseline hypoalbuminemia. Importantly, albumin levels were measured at admission to capture acute inflammatory and hemodynamic stress rather than long-term nutritional status. The Mendelian randomization study by Huang et al. further suggested that genetically lower albumin levels may predispose individuals to PE [9], although this relationship may vary across populations.

The relationship between albumin levels and clinical severity was evident, with a moderate, statistically significant inverse correlation between albumin levels and PESI scores (r = -0.382, p < 0.001). This finding indicates that lower albumin concentrations are consistently associated with higher PESI classes and a higher predicted mortality risk. Albumin does not appear to be a marker of only acute hemodynamic compromise but rather reflects general physiological vulnerability, including systemic inflammation, endothelial dysfunction, and chronic disease burden, which are associated with worsening PESI classification. Albumin showed poor discriminatory performance in the ROC analysis (AUC = 0.628), but an optimal cutoff of 31.9 g/L yielded high sensitivity and moderate specificity. Our findings are in line with growing evidence for albumin-based composite indices, including the CRP/Alb ratio [12], platelet-to-albumin ratio [13], RDW/Alb ratio [14,15], and RDW-SD/Alb ratio [16], which have demonstrated enhanced prognostic accuracy in acute PE. However, we did not investigate these indices in this study. Our results indicated that albumin may be a useful component in multiparameter risk stratification models.

Beyond albumin level, several clinical factors independently predicted massive PE, including hemodynamic instability, bradycardia, right ventricular strain, and prior stroke. These results are consistent with the known pathophysiology of PE in which acute right ventricular failure, decreased cardiac output, and systemic hypoperfusion cause clinical deterioration [1,2]. This strong relationship between right ventricular strain and massive PE is consistent with the findings of previous studies that identified right ventricular dysfunction as an important prognostic marker [4-6]. A previous stroke and massive PE may reflect increased immobility, chronic inflammation, or challenges in anticoagulation management. Differences in the reported associations are likely due to differences in comorbidity profiles and stroke-related disabilities across the studies.

Overall, these results confirm the multifactorial nature of PE severity and the possible usefulness of serum albumin alone and as a composite index for risk stratification. The addition of albumin to clinical, hemodynamic, and echocardiographic parameters may assist in the early identification of high-risk patients, especially in settings where more sophisticated PE response systems are not available.

Study limitations

This study had limitations that may affect the generalizability of the findings. First, its retrospective design precluded the establishment of causal relationships. Second, the relatively small number of patients with massive PE (N = 25) may have reduced the statistical precision and widened the CIs. Third, because this was a single-center study, the findings may not be generalizable to other populations with different demographic and clinical characteristics. Finally, important confounders such as inflammatory markers, liver function tests, and long-term nutritional status were not evaluated, which may have affected the observed association between albumin levels and PE severity.

## Conclusions

Serum albumin levels have emerged as an important biochemical marker linked to PE severity, massive PE, and mortality in this cohort. Low albumin levels independently predicted massive PE, indicating that even modest reductions in albumin levels may signal heightened physiological vulnerability and a greater likelihood of hemodynamic compromise. Albumin also demonstrated a significant inverse correlation with PESI score in the overall cohort, supporting its role as a marker of global clinical severity. Low albumin levels were significantly associated with in-hospital mortality, reinforcing their relevance as a prognostic indicator. ROC analysis further showed that albumin had a moderate discriminatory ability for identifying massive PE, with high sensitivity at the optimal cutoff. Overall, these findings indicate that albumin is a more informative marker when incorporated into multiparameter risk assessment strategies, particularly alongside hemodynamic instability, right ventricular strain, bradycardia, and prior stroke, rather than as a standalone marker. Larger multicenter studies are needed to validate these observations and clarify the mechanistic pathways linking albumin levels to PE severity and outcomes.
